# Supplementary material for: Experimental Infection of Brazilian Free-Tailed Bats (Tadarida brasiliensis) with Two Strains of SARS-CoV-2
Source: Viruses. 2022 Aug 18;14(8):1809. doi: 10.3390/v14081809 (PMC9412320; doi:10.3390/v14081809)
Supplement: Supplementary file 1 [file viruses-14-01809-s001.zip › viruses-1810045-supplementary.pdf]

Supplemental Table S1: Complete histopathological findings and scoring system for control and inoculated bats

**Lesion**	**#control**	**#treatment**	**Interpretation**	**Additional Details on Treatment Bats with Lesion**
Prominent bronchus-associated lymphoid tissue (BALT)	2/6	14/18	Could suggest general immune stimulation	8/9 Delta Bats, 6/9 WA01 Bats
Splenic lymphoid hyperplasia	0/6	7/18	Could suggest general immune stimulation	5/9 Delta, 2/9 WA01 Bats
Stomatitis, erosive to ulcerative	0/6	3/18	Suspected irritation from swabbing	Delta Bat 4 (Delta), Delta Bat 5 (Delta), and Delta Bat 9 (Delta) all survived to planned euthanasia time points. Bats Delta 4 and Delta 5 also had non-negative Ct values for lung PCR
Interstitial pneumonia	3/6	1/18	Control bats all died or were euthanized within 14 days of capture. Results in control bats were consistent with ill thrift, poor intakes, parasitism, and bacteremia. No apparent association with SARS-CoV-2 infection	Bat 1B (WA01) survived until 28 dpi. This bat performed well in captivity and had abundant fat stores at the time of euthanasia
Tracheitis/pharyngitis, mild, suppurative	1/6	0/18	Control bats all died or were euthanized within 14 days of capture. Results are consistent with ill thrift, poor intakes, parasitism, and bacteremia	n/a
Splenic lymphoid depletion	0/6	1/18	Ill thrift	Bat 2 (WA01) died at 3 dpi due to ill thrift
Hepatocellular necrosis, multifocal, random, with multifocal suppurative hepatitis	0/6	1/18	Ill thrift	Delta Bat 6 (Delta) was found dead 14 dpi due to ill thrift

Nasal turbinates, intraluminal mucinous debris	0/6	2/18	Findings likely attributed to ill thrift and secondary infections	Delta Bat 3 (Delta) was found dead 14 dpi due to ill thrift. Delta Bat 6 (Delta) was found dead 14 dpi due to ill thrift
Gastric erosions with bile staining of mucosa	3/6	1/18	Low intakes. Affected bats all died or were euthanized within (x) days of capture. Results are consistent with ill thrift, poor intakes, parasitism, and bacteremia	Delta Bat 3 (Delta) was found dead 14 dpi due to ill thrift
Intestinal coccidiosis	1/6	1/18	Parastism common in free-ranging bats	Bat 2 (WA01) died at 3 dpi due to ill thrift
Intestinal nematodiasis	1/6	0/18	Parastism common in free-ranging bats	n/a
Biliary trematodiasis	1/6	0/18	Parasitism common in free-ranging bats	n/a
Colonic trematodiasis	1/6	0/18	Parasitism common in free-ranging bats	n/a
Gastritis, ulcerative multifocal, +/- intraluminal trematode	2/6	2/18	Parastism common in free-ranging bats	Bat 7 (WA01) was found dead 2 dpi due to ill thrift Delta Bat 6 (Delta) was found 14 dpi due to ill thrift
Intestinal trematodiasis	0/6	2/18	Parasitism common in free-ranging bats	Bat 7 (WA01) was found dead 2 dpi due to ill thrift. Bat 3B (WA01) survived until 28 dpi. This bat performed well in captivity and had abundant fat stores at the time of euthanasia and hydropic degeneration in the liver. No lesions were observed in other organs.
Hepatic centrilobular to panlobular hydropic degeneration	0/6	3/18	Findings are consistent with excessive fat stores	These bats performed well in captivity and had abundant fat stores at the time of euthanasia and hydropic degeneration in the liver. No lesions were observed in other organs.
Abundant subcutaneous adipose tissue/obesity	0/6	5/18	Findings are consistent with excessive fat stores	These bats performed well in captivity and had abundant fat stores at the time of euthanasia +/- hydropic degeneration in the liver. No lesions were observed in other organs.

Supplemental Table S2: Bat Oral Swab PCR CT values.

**Bat ID**	**1 DPI**	**2 DPI**	**3 DPI**	**4 DPI**	**5 DPI**	**7 DPI**
Bat 1	32.507	32.363	30.172	NT	NT	NT
Bat 2	33.419	34.093	31.302	NT	NT	NT
Bat 3	27.102	35.131	33.234	NT	NT	NT
Bat 5	Undetermined	32.832	34.018	32.328	31.765	32.312
Bat 6	30.099	32.370	29.341	NT	NT	NT
Bat 7	31.167	33.077	NT	NT	NT	NT
Bat 8	32.030	31.476	35.079	32.798	32.277	30.577
Bat 9	29.770	33.619	32.535	32.778	32.799	31.357
Bat 10	32.593	33.435	32.558	33.229	32.919	31.628
Bat 1D	32.766	32.405	33.908	34.003	34.356	35.941
Bat 2D	32.735	34.224	33.642	33.624	34.436	33.578
Bat 3D	Undetermined	34.851	35.778	Undetermined	34.315	34.549
Bat 4D	31.682	32.769	33.409	NT	NT	NT
Bat 5D	Undetermined	34.400	35.912	34.842	33.854	34.773
Bat 6D	34.667	32.731	34.813	37.080	Undetermined	35.216
Bat 7D	34.047	32.290	32.808	NT	NT	NT
Bat 8D	32.471	32.854	40.866	NT	NT	NT
Bat 9D	39.327	32.888	36.100	33.217	37.305	31.750

NT = Not tested
